# Supplementary material for: Water Quality Evaluation and Pollution Source Apportionment of Surface Water in a Major City in Southeast China Using Multi-Statistical Analyses and Machine Learning Models
Source: Int J Environ Res Public Health. 2023 Jan 3;20(1):881. doi: 10.3390/ijerph20010881 (PMC9820299; doi:10.3390/ijerph20010881)
Supplement: Supplementary file 1 [file ijerph-20-00881-s001.zip › ijerph-2055558-supplementary.pdf]

Supplementary Materials

Water quality evaluation and pollution source apportionment of surface water in a major city in southeast China using multi-statistical analyses and machine learning models

Yu Zhou[1], Xinmin Wang[1], Weiying Li[2, 3, *], Shuyun Zhou[4] and Laizhu Jiang[5]

[1] College of Environmental Science and Engineering, Tongji University, Shanghai, 200092, China;
[2] State Key Laboratory of Pollution Control and Resource Reuse, Tongji University, Shanghai, 200092, China;
[3] Ministry of Education Key Laboratory of Yangtze River Water Environment, Tongji University, Shanghai, 200092, China;
[4] Jiangsu Yinyang Stainless Steel Pipe Co., Ltd;
[5] Fujian Qingtuo Special Steel Technology Research Co., Ltd;
* Correspondence: 123lwyktz@tognji.edu.cn; Tel.: 18017982085

Table S1. Water quality characteristic

Parameters	Site	N	Max	Min	Mean	SD	CV	National surface water quality standard (GB 3838-2002)						Exceeding standard Ⅲ rate (%)
									I	II	Ⅲ	Ⅳ	V	
WT (℃)	FWP	44	28.1	11.8	21.59	3.93	18.18	No available standard						/
	WWP	119	29	11.5	21.46	4.53	21.13							/
	SWP	97	30.6	11.1	21.58	4.87	22.59							/
	CWP	119	30.4	11.1	21.71	4.79	22.08							/
TP (mg/L)	FWP	44	0.08	0.01	0.02	0.02	74.11	≤	0.02	0.1	0.2	0.3	0.4	0
	WWP	119	0.2	0.01	0.03	0.03	106.41							0
	SWP	97	0.31	0.01	0.05	0.04	92.22							0
	CWP	119	0.14	0.01	0.04	0.03	84.12							1.02
TN (mg/L)	FWP	44	2.19	0.49	1.42	0.45	31.4	≤	0.2	0.5	1	1.5	2	82.35
	WWP	119	3.36	0.14	1.53	0.58	37.73							77.27
	SWP	97	4.76	0.2	1.64	0.78	47.54							89.92
	CWP	119	3.48	0.26	1.64	0.57	34.94							75.64
SO_4^{2-} (mg/L)	FWP	44	28.5	2.99	14	6.48	46.32	≤	250					0
	WWP	119	27.2	4.95	13.05	4.84	37.07							0
	SWP	97	20.37	2.99	9.24	4.38	47.35							0
	CWP	119	56	2.76	14.1	7.52	53.36							0
pH	FWP	44	7.49	6.58	6.97	0.19	2.68		6~9					0
	WWP	119	7.37	6.53	6.92	0.17	2.47							0
	SWP	97	7.33	6.58	6.93	0.14	2.08							0
	CWP	119	7.6	6.5	7.01	0.2	2.91							0
NO_3^- (mg/L)	FWP	44	2.37	0.5	1.33	0.41	30.97	≤	10					0
	WWP	119	3.55	0.74	1.6	0.46	29.15							0
	SWP	97	4.07	0.55	1.6	0.6	37.21							0
	CWP	119	3.38	0.66	1.61	0.5	31.24							0
Mn (mg/L)	FWP	44	0.13	0	0.05	0.04	70.04	≤	0.1					6.72

Parameters	Site	N	Max	Min	Mean	SD	CV	National surface water quality standard (GB 3838-2002)						Exceeding standard Ⅲ rate (%)
									I	II	Ⅲ	Ⅳ	V	
	WWP	119	0.16	0	0.06	0.03	47.42							11.36
	SWP	97	1.52	0	0.09	0.16	82.77							44.54
	CWP	119	0.55	0	0.12	0.09	80.38							23.08
Fe (mg/L)	FWP	44	1.37	0.01	0.26	0.21	81.87	≤			0.3			21.01
	WWP	119	1.26	0	0.28	0.14	50.96							11.36
	SWP	97	2.63	0	0.37	0.32	88.36							44.54
	CWP	119	3.2	0.01	0.76	0.56	73.93							23.08
F.coli (colonies/L)	FWP	44	32000	210	4576	6527	142.64	≤	200	2000	10000	20000	40000	21.01
	WWP	119	65000	600	7553	8909	117.94							9.09
	SWP	97	160000	10	22031	24295	110.28							51.26
	CWP	119	92000	100	14115	13107	92.86							53.85
F^- (mg/L)	FWP	44	0.38	0.13	0.23	0.06	24.84	≤	1	1	1	1.5	1.5	0
	WWP	119	0.43	0.14	0.25	0.06	23.3							0
	SWP	82	0.48	0.1	0.22	0.08	36.77							0
	CWP	119	0.45	0.03	0.26	0.07	26.18							0
DO (mg/L)	FWP	44	9.92	4.2	7.19	1.2	16.7	≥	7.5	6	5	3	2	3.36
	WWP	119	10.7	3.39	7.42	1.41	19.06							2.27
	SWP	82	10.8	3.65	6.77	1.6	23.69							5.04
	CWP	119	10.5	3.51	7.21	1.34	18.55							10.26
COD_{Mn} (mg/L)	FWP	44	4.38	1.01	1.75	0.61	34.92	≤	2	4	6	10	15	0
	WWP	119	3.58	0.64	1.79	0.53	29.52							0
	SWP	82	3.58	0.4	2.03	0.69	34.04							0.84
	CWP	119	7.75	0.56	2.13	0.79	36.96							0
Cl^- (mg/L)	FWP	44	11.5	1.46	5.09	2.36	46.42	≤			250			0
	WWP	119	11.2	1.6	5.53	2.04	36.87							0
	SWP	82	12.58	1.89	5.14	2.49	48.43							0.84
	CWP	119	253	1.82	13.77	31.44	228.23							0

Parameters	Site	N	Max	Min	Mean	SD	CV	National surface water quality standard (GB 3838-2002)						Exceeding standard Ⅲ rate (%)
									Ⅰ	Ⅱ	Ⅲ	Ⅳ	Ⅴ	
NH_3-N (mg/L)	FWP	44	0.16	0.02	0.04	0.04	94.56	≤	0.15	0.5	1	1.5	2	0
	WWP	119	0.57	0.02	0.09	0.1	121.01							0
	SWP	82	0.45	0.02	0.1	0.1	107.85							0
	CWP	119	0.57	0.02	0.13	0.13	97.18							0

N number of samples, SD standard deviation, CV coefficient of variation

Table S2. Weights and normalization factors of the parameters used in the calculation of the water

**Abbreviation**	**Units**	**Relative weight (pi)**	**Normalization factor (Ci)**										
			**100**	**90**	**80**	**70**	**60**	**50**	**40**	**30**	**20**	**10**	**0**
T	°C	1	22/15	24/14	26/12	28/10	30/5	32/0	36/−2	40/−4	45/−6	>45/<−6	>45/<−6
pH		1	7	7-8	7-8.5	7-9	6.5-7	6-9.5	5-10	4-11	3-12	2-13	1-14
DO	mg/L	4	≥7.5	>7	>6.5	>6	>5	>4	>3.5	>3	>2	≥1	<1
TN_a	mg/L	2	<0.1	<0.2	<0.35	<0.5	<0.75	<1	<1.25	<1.5	<1.75	≤2	>2
NH_4-N	mg/L	3	<0.01	<0.05	<0.1	<0.2	<0.3	<0.4	<0.5	<0.75	<1	≤1.25	>1.25
NO_3-N_b	mg/L	2	<0.5	<2	<4	<6	<8	<10	<15	<20	<50	≤100	>100
TP_a	mg/L	1	<0.01	<0.02	<0.05	<0.1	<0.15	<0.2	<0.25	<0.3	<0.35	≤0.4	>1.00
COD_{Mnc}	mg/L	3	<1	<2	<3	<4	<6	<8	<10	<12	<14	≤15	>15
Cl^-	mg/L	1	<25	<50	<100	<150	<200	<300	<500	<700	<1000	≤1500	>1500
SO_4^{2-}	mg/L	2	<25	<50	<75	<100	<150	<250	<400	<600	<1000	≤1500	>1500
F.coli	colonies/L	3	<50	<100	<200	<1000	<2000	<6000	<10000	<15000	<20000	≤400000	>40000
Fe	mg/L	3	<0.03	<0.06	<0.10	<0.2	<0.3	<0.4	<0.5	<0.75	<1	≤1.5	>1.5
Mn	mg/L	3	<0.01	<0.02	<0.035	<0.05	<0.075	<0.1	<0.125	<0.15	<0.2	≤0.3	>0.3
F^-	mg/L	2	<0.1	<0.2	<0.35	<0.5	<0.75	<1	<1.25	<1.5	<1.75	≤2	>2

Adopted from Koçer and Sevgili[1], Wu et al.[2], Nong et al.[3], and Pak et al. [4]

Table S3. The parameter selection results of the WQI_{LRmin} models from the stepwise multiple linear regression

Parameters	R^2	MSE	MAE	MAPE/%
Mn	0.583	16.919	3.391	4.802
Mn + Fe	0.677	14.135	3.047	4.317
Mn + Fe + *F. coli*	0.766	9.500	2.448	3.499
Mn + Fe + *F. coli* + DO	0.865	5.496	1.868	2.715
Mn + Fe + *F. coli* + DO + TN	0.905	2.527	1.232	1.728

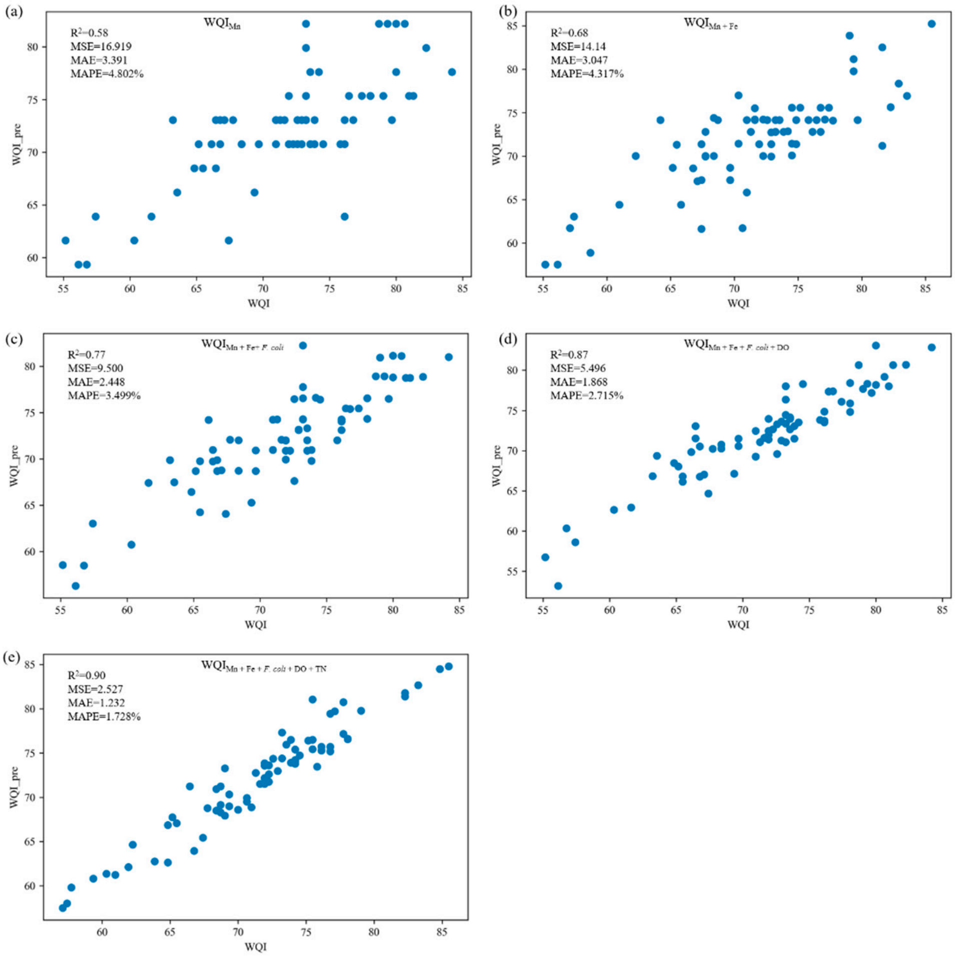

Figure. S1. Comparison of the WQI and WQI_{LRmin} values from the stepwise multiple linear regression based on the testing dataset

References

1. Kocer, M.; Sevgili, H. Parameters selection for water quality index in the assessment of the environmental impacts of land-based trout farms. Ecol Indic 2014, 36, 672-681, doi:10.1016/j.ecolind.2013.09.034.
2. Wu, Z.S.; Wang, X.L.; Chen, Y.W.; Cai, Y.J.; Deng, J.C. Assessing river water quality using water quality index in Lake Taihu Basin, China. Sci Total Environ 2018, 612, 914-922, doi:10.1016/j.scitotenv.2017.08.293.

3. Nong, X.Z.; Shao, D.G.; Zhong, H.; Liang, J.K. Evaluation of water quality in the South-to-North Water Diversion Project of China using the water quality index (WQI) method. Water Res 2020, 178, doi:10.1016/j.watres.2020.115781.
4. Pak, H.Y.; Chuah, C.J.; Tan, M.L.; Yong, E.L.; Snyder, S.A. A framework for assessing the adequacy of Water Quality Index - Quantifying parameter sensitivity and uncertainties in missing values distribution. Sci Total Environ 2021, 751, doi:10.1016/j.scitotenv.2020.141982.
